# A Brief Notice of the Treatment of Dysentery; the Honey Bee as a Diuretic; and Warm Water in Colic

**Published:** 1851-08

**Authors:** M. H. Fee

**Affiliations:** Heltonville, Indiana


Art. III.—*A brief notice of the Treatment of Dysentery; the Honey Bee as a Diuretic; and Warm Water in Colic.* By Dr. M. H. Fee, of Heltonville, Indiana.

I herewith communicate to you a few facts, which you are at liberty to put to any use you please. If you think them of any value, you may give them publicity in your Journal, under the head of "Facts tested by experience."

The last summer was fruitful, with us, in acute dysentery, mostly of that form, known among the people, by the name of "gray flux," that is, mostly mucus mixed with blood constituting the alvine discharges. Condie's treatment of calomel, chalk, camphor, hyosciamus, and sugar of lead proved worse than useless; constantly increasing the tormina, drying the tongue, and increasing the soreness of the abdomen. Being left to my own resources, I struck out the following prescription, which was eminently successful, not failing in any case:

℞. Calomel, grs. v;
Camphor, grs. v;
Ipecac., grs. ii;
Dover's powder, grs. v; every three hours till five doses were given.

The result of this treatment was free perspiration, nausea, emesis, and perfect relaxation of spasm and tormina; large, free, bilious stools; in short, convalescence was immediately established. I believe this prescription would scarcely fail to effect a cure once in a hundred cases.

*The Honey Bee an Anti-Cantharidic and Diuretic.*—Three or four years ago I saw it stated in your journal, by Dr. F. H. Gordon, of Tennessee, that an infusion of honey bees would generally relieve strangury from a blister in fifteen minutes. I resolved to try it the first opportunity. In a short time I had a chance for the trial of the virtues of the infusion, and was gratified to find it prompt. Fifteen minutes are generally sufficient for the display of its effects. I find that it is applicable still further. In those cases of urinary suspension from a too liberal use of opium, it cannot be too highly lauded. It is a bland diuretic, increasing the secretion of the kidneys, at the same time quieting too great excitability of those organs. It may be used with decided advantage in lithiasis, attended by deposition of lithic acid crystals; as also in painful micturition from the terebinthinates.

℞. 60 honey bees;
Boiling water, 1 pint; cover and draw 15 minutes; dose, one gill every 15 minutes.

The above prescriptions are for adults.

*Warm Water in Colic.*—The best remedy I have ever tried for spasmodic colic, and for that incessant disposition to vomit in fall congestive fevers, is warm water, taken in copious draughts every fifteen minutes till the stomach and bowels relax to their natural caliber, and the skin becomes moist.

March 11, 1851.

[*Note, by the Editors.*—It will be remarked how confidently Dr. Fee speaks of the powers of his prescription in dysentery. A medical friend has just informed us, that he has a combination which he regards as a *specific* in that disease; namely, sulphur ʒi, charcoal ʒij; to be given every two or three hours. He assures us that quinine is not a more certain remedy for intermittent fever than he has found this formula to be in dysentery. Now, it is a circumstance to shake our confidence in these specifics that they have become so much multiplied. We do not, for a moment, question the correctness of the reports of our friends; but as to the efficacy of their prescriptions—will they stand the test of experience? Is it not possible that the cases of dysentery in which they proved so effectual were mild cases? At all events, it is not a little calculated to excite distrust in the value of treatment in any disease, that so many plans are extolled as sovereign, and soon again abandoned as ineffectual.]

